# Moderate Light Intensity Optimizes Forage Nutritive Value While Maintaining Morphophysiological Stability and Secondary Metabolite Concentrations in *Plantago lanceolata* L. Under Controlled Environmental Conditions

**DOI:** 10.3390/plants15081274

**Published:** 2026-04-21

**Authors:** Verónica M. Merino, Luis F. Piña, M. Jordana Rivero, Neal B. Stolpe, Luisa L. Bascuñán, Pablo A. Castro, José M. Ortiz, María D. López, Gabriela E. Gómez, Baska R. Concha

**Affiliations:** 1 Departamento de Producción Animal, Facultad de Ciencias Agronómicas, Universidad de Chile, Santiago 8820808, Chile; 2 Laboratorio de Nutrición Animal, Departamento de Producción Animal, Facultad de Ciencias Agronómicas, Universidad de Chile, Santiago 8820808, Chile; gaby.gomez.lillo@gmail.com; 3 Farming Footprints and Adaptations, Rothamsted Research, Okehampton EX20 2SB, UK; jordana.rivero.visiting@rothamsted.ac.uk; 4 Departamento de Suelos y Recursos Naturales, Facultad de Agronomía, Universidad de Concepción, Casilla 160-C, Concepción 4030000, Chile; nstolpe@udec.cl; 5 Departamento de Botánica, Facultad de Ciencias Naturales y Oceanográficas, Universidad de Concepción, Concepción 4030000, Chile; lubascun@udec.cl (L.L.B.); pabcastro@udec.cl (P.A.C.); joseortizg@udec.cl (J.M.O.); 6 Departamento de Producción Vegetal, Facultad de Agronomía, Universidad de Concepción, Av. Vicente Méndez 595, Chillán 3780000, Chile; mlopezb@udec.cl; 7 Universidad de Concepción, Concepción 4030000, Chile; bconcha2018@udec.cl

**Keywords:** plantain, photosynthesis, morphological characteristics, nutritional value, forage, pasture

## Abstract

*Plantago lanceolata* L. is increasingly incorporated in temperate pasture systems for its agronomic resilience and potential to reduce the environmental footprint of ruminant production through its specific secondary metabolites (SMs). However, how light intensity per se regulates *P. lanceolata* L. physiology, nutritive value and SM accumulation remains poorly understood due to confounding factors in field studies. This controlled-environment study evaluated the effects of three light intensities (200, 300, and 400 µmol photons m^−2^ s^−1^) on morphophysiological traits, forage quality, and SM concentrations in *P. lanceolata* L. cv. “Ceres Tonic”. Plants were grown in controlled-environment chambers under similar temperature, humidity and nutrient conditions. Morphological traits, biomass allocation, chlorophyll fluorescence, gas exchange, chemical composition, and root architecture were measured. Additionally, the most important secondary metabolites, aucubin, catalpol and acteoside, were also evaluated. Under the different light intensity treatments plants maintained stable physiological parameters, total biomass production, leaf dimensions or root architecture. However, moderate light intensity (300 µmol photons m^−2^ s^−1^) optimized nutritive value by minimizing fiber concentrations and maximizing metabolizable energy. Acteoside concentration, as well as the iridoid glycosides aucubin and catalpol, were not affected by the different light intensities. These findings demonstrate that *P. lanceolata* L. maintains morphophysiological stability across the tested light intensity range studied, while selectively modulating forage quality.

## 1. Introduction

*Plantago lanceolata* L. (plantain) has emerged as a strategic functional species in temperate pasture-based dairy and livestock systems, particularly under increasing climatic variability [1]. Compared to traditional perennial ryegrass (*Lolium perenne* L.), *P. lanceolata* L. exhibits superior adaptability to warm and dry conditions, contributing to enhanced seasonal stability of herbage dry matter (DM) production and nutritional value [2,3]. Its deep and extensive root system supports growth during summer and autumn, periods when conventional ryegrass-based pastures commonly exhibit marked declines in productivity and forage quality [4]. Beyond its agronomic advantages, *P. lanceolata* L. provides important environmental co-benefits by reducing urinary nitrogen (N) excretion and mitigating N leaching and nitrous oxide (N_2_O) emissions in pastoral systems [5,6,7]. Furthermore, consistent reductions in methane (CH_4_) emissions from ruminants grazing *P. lanceolata* pastures have been documented [8,9]. These antimethanogenic effects have been partly attributed to the direct activity of the principal SM of *P. lanceolata* L. on ruminal fermentation. Sivanandarajah et al. (2025) [5] demonstrated *in vitro* that acteoside, aucubin and catalpol inhibit methanogenic archaea in a concentration-dependent manner, establishing a direct mechanistic link between individual SM concentrations in *P. lanceolata* L. herbage and its capacity to reduce enteric CH_4_ production, positioning *P. lanceolata* L. not only as a drought-tolerant species but also as a natural strategy for reducing the environmental footprint of pasture-based livestock production systems.

The capacity of *P. lanceolata* L. to reduce N losses from grazed pastures has been attributed to its unique profile of secondary metabolites (SMs), particularly the iridoid glycosides aucubin and catalpol, and the phenylethanoid glycoside acteoside, also known as verbascoside, which influence N dynamics during ruminal fermentation, thereby contributing to reduced urinary N output [10]. However, the concentrations of these SMs in *P. lanceolata* L. are highly variable and are influenced by genotype [11], plant phenological state [12,13], nutrient supply and environmental growth conditions [14]. Among environmental drivers, light availability has been identified as a key factor affecting both primary metabolism and the biosynthesis of SMs in *P. lanceolata* L., particularly aucubin and acteoside concentrations [14,15].

Field studies have reported strong seasonal variation in aucubin, catalpol and acteoside concentrations, often positively associated with light intensity and solar radiation. Miehe-Steier et al. (2015) [14] reported that high light significantly increased iridoid glycosides and acteoside concentrations compared to low light in pastures in wild populations of *P. lanceolata* L., reflecting considerable phenotypic plasticity, whilst Box et al. (2019) [16] documented substantial seasonal variation in acteoside concentrations in the “Ceres Tonic” cultivar with strong positive associations with solar radiation. Similarly, aucubin concentrations in “Ceres Tonic” *P. lanceolata* L. cultivar vary substantially over the growing season [10,13]. However, these field-based studies involved multiple confounding environmental variables such as temperature, photoperiod, water availability and nutrient status, making it difficult to isolate the specific effects of light intensity on SM biosynthesis and associated productive and morpho-physiological responses.

Despite the demonstrated importance of *P. lanceolata* L. SMs in reducing the environmental footprint of grazing systems, fundamental knowledge gaps remain regarding how light intensity, as an isolated environmental factor, regulates their biosynthesis. Furthermore, the integrated effects of light intensity on photosynthetic performance, nutritional quality, morphological traits, biomass distribution and SM concentrations under controlled environmental conditions have not been comprehensively characterized. Understanding how light intensity per se affects *P. lanceolata* L. physiology, nutritional quality and secondary metabolite concentrations under controlled conditions is a necessary mechanistic step toward interpreting the more complex, multi-factor responses observed in field pastures, and provides a scientific basis for evaluating whether light-modifying management practices—such as adjusting sward density, grazing height, or companion species composition—may influence forage quality. The objective of this study was to assess, under controlled environmental conditions and repeated defoliation to simulate grazing pressure in forage systems, the effects of three levels of light intensities (200, 300, and 400 µmol photons m^−2^ s^−1^; L200, L300 and L400, respectively) on growth performance, morpho-physiological traits, forage nutritive value and concentrations of SMs in *P. lanceolata* L. cv. “Ceres Tonic”. We hypothesized that moderate light intensity would optimize forage quality, while higher light intensity would enhance photoprotective responses and increase the SMs content.

## 2. Results

### 2.1. Leaf Characteristics and Morphological Characteristics Across Each Cut

The length and width of the extended leaf did not vary with light intensity, averaging 19.3 ± 0.70 cm (*p* = 0.702) and 2.59 ± 0.425 cm (*p* = 0.250), respectively. None of the morphological characteristics, biomass composition and herbage biomass production at each cut made at 5 cm of residual height was affected by the light intensity treatments. The number of total leaves per pot averaged 70.8 ± 9.05 (*p* = 0.336), the number of reproductive stems per pot was 33.7 ± 2.32 (*p* = 0.089; L400 tended to have ~8 reproductive stems less than the other two groups), the number of total leaves per plant was 17.7 ± 0.19 (*p* = 0.324), the number of mature leaves per plant was 14.4 ± 1.98 (*p* = 0.325), the number of immature leaves per plant was 3.32 ± 0.36 (*p* = 0.444), the number of dead leaves per plant was 1.38 ± 0.190 (*p* = 0.226), the number of reproductive stems per plant was 8.42 ± 0.579 (*p* = 0.089; L400 tended to have ~2 more reproductive stems per plant than the other two groups), the total DM weight per cut per pot was 12.2 ± 0.58 g (*p* = 0.675), the leaves dry mass per pot was 5.85 ± 0.668 g (*p* = 0.605), the reproductive stems dry mass per pot was 5.76 ± 0.362 g (*p* = 0.713), the dead dry mass per pot was 0.548 ± 0.0952 g (*p* = 0.194), the leaf DM weight as % total DM was 54.3 ± 2.96% (*p* = 0.094; it tended to decrease from L200 to L400), the reproductive stem DM weight as % total DM was 43.2 ± 2.89% (*p* = 0.139), and the dead material DM weight as % DM was 2.45 ± 0.65% (*p* = 0.687).

### 2.2. Effects of Light Intensity on Chlorophyll Fluorescence Parameters

The only physiological variables affected by the light intensity were the F_0_ (minimum chlorophyll fluorescence), F_m_ (maximum chlorophyll fluorescence) and F_v_/F_m_ (maximum quantum yield of PSII) of mature leaves (Table 1). F_0_ was highest in L200 and lowest in L300 (*p* = 0.027). Similarly, F_m_ was highest in L200 and L300 and lowest in L400 (*p* = 0.001). The F_v_/F_m_ of mature leaves followed the same pattern, with highest values in L200 and L300 and the lowest for L400 (*p* = 0.030) (Table 1). For immature leaves, all fluorescence parameters were unaffected by light intensity; F_0_ averaged 94.1, F_m_ averaged 659, and F_v_/F_m_ averaged 0.856. Net photosynthetic rate, stomatal conductance (gs) and apparent transpiration rate (E) did not differ amongst treatments for either leaf type (Table 1). Interestingly, immature leaves showed similar An values to mature leaves. This indicates a robust photosynthetic apparatus, which is also supported by the stability of fluorescence parameters including F_0_, F_m_ and F_v_/F_m_, which showed only slight changes in response to light and remained within ranges indicative of healthy physiological status, indicating no photodamage under any light condition.

Regarding the relationship between net CO_2_ assimilation (An) and stomatal conductance (gs), we found that mature leaves under L200 exhibited no significant correlation (r = −0.34, *p* = 0.42), in contrast to all other combinations of light intensity and leaf developmental stage, which showed strong positive correlations (r > 0.70, *p* ≤ 0.05; Table 2). Notably, immature leaves maintained a strong positive relationship between An and gs across all light treatments, including under low light conditions (r = 0.72, *p* = 0.045).

### 2.3. Effects of Light Intensity on Forage Nutritive Value

Regarding nutritional composition, the only variables affected by light intensity were fiber and ME concentrations (Table 3). DM concentration tended (*p* = 0.0615) to be greater in L300 and averaged 23.6 g/100 g of fresh matter (FM). CP tended to be greater in L400 (*p* = 0.0671) and averaged 9.29% DM. NDF was the highest for L400 and the lowest for L300, while ADF was lowest for L300 and highest for the other treatments. Inversely, ME was the highest for L300 and the lowest for other treatments. Ash concentration averaged 10.0%. Total carbohydrates averaged 147 mg/100 g, low-WSC averaged 113 mg/100 g and high-WSC averaged 33.2 mg/100 g (Table 3).

### 2.4. Destructive Harvest

Leaf area did not vary with light intensity: mature leaves averaged 29.2 ± 1.95 cm^2^ (*p* = 0.343) and immature leaves 12.7 ± 1.20 cm^2^ (*p* = 0.372). The number of immature leaves per plant was the greatest in L300 and the lowest in L200 (*p* = 0.005) (Table 4). The dry mass of the immature leaves was the highest in L300 and L400 (*p* = 0.001) while the dry mass of the senescent leaves was the highest in L300 and the lowest in L200 (*p* = 0.048) (Table 5). None of the other variables measured in the destructive harvest were affected by the light intensity, although some tended to vary (Table 5); all the overall averages of these variables are presented in Table 4 and Table 5.

### 2.5. Secondary Metabolites

None of the SMs analyzed varied with the light intensity treatment, neither in immature nor mature leaves (Kruskal–Wallis H statistic *p* > 0.05). However, the acteoside concentration in the immature leaves tended (Kruskal–Wallis H statistic *p* = 0.064) to show a positive trend with increasing light intensity, being 5.67 mg g^−1^ DM for L200, 10.0 mg g^−1^ DM for L300 and 12.8 for L400.

### 2.6. Root Traits

Root traits did not differ among treatments (*p* > 0.05). Total root length averaged 5389 cm (*p* = 0.554), total surface area averaged 684 cm^2^ (*p* = 0.502), root diameter averaged 0.406 mm (*p* = 0.366) and root volume averaged 6.99 cm^3^ (*p* = 0.386). The total number of crossings averaged 6968 (*p* = 0.766), the number of forks averaged 33,506 (*p* = 0.541) and the number of tips averaged 12,468 (*p* = 0.158).

## 3. Discussion

This study provides novel mechanistic insights into how light intensity, isolated from confounding environmental variables, regulates the interplay between leaf dimensions, herbage production, nutritional quality, photosynthetic function and SM content in *P. lanceolata* L. Our controlled-environment study demonstrates that light intensity affects nutritional quality and photosynthetic performance in *P. lanceolata* L. Moderate light intensity (300 µmol photons m^−2^ s^−1^) optimized forage quality by minimizing fiber content and maximizing metabolizable energy, whilst higher light intensity (400 µmol photons m^−2^ s^−1^) induced photosynthetic subtle adjustments in chlorophyll fluorescence parameters of mature leaves, while no significant effects on secondary metabolite concentrations were detected. These results provide novel insights into the environmental regulation of *P. lanceolata*’s nutritional value, with direct implications for optimizing pasture management strategies under different climate change scenarios.

### 3.1. Productive and Morphological Responses and Phenotypic Plasticity

The relatively limited morphological responses to light intensity observed in our study, with leaf dimensions, herbage biomass production, DM allocation patterns, root architectural traits and most morphological characteristics and composition remaining statistically invariant, contrast with the substantial morphological plasticity documented in natural ecotypes of *P. lanceolata* L. populations grown under different light quality regimes [14]. These findings indicate remarkable morphological stability in *P. lanceolata* L. cv. “Ceres Tonic” when exposed to moderate variations in light intensity under controlled environmental conditions. Moreover, this apparent discrepancy can be reconciled by recognizing the fundamental distinction between light intensity effects and light quality (spectral composition) effects on plant morphogenesis. Van Tienderen and Van Hinsberg (1996) [17] demonstrated that in *P. lanceolata* L., light intensity primarily affects plant size, whereas light quality, particularly the red:far-red (R:FR) ratio, affects growth habit and architectural traits such as petiole length, leaf angle, and rosette compactness. Van Hinsberg and Van Tienderen (1997) [18] further showed that sun-adapted populations produce prostrate rosettes with short leaves, while shade-adapted populations produce long, erect leaves, with low R:FR promoting longer, more upright leaves and increased allocation to shoot growth. These morphological adjustments represent classic shade-avoidance responses mediated by phytochrome signaling and gibberellin biosynthesis [19]. In our study, all three light treatments were provided using identical LED panels with a constant spectral composition (68% red, 12% blue, 14% white, 4% far-red, 2% UV) across all treatments, differing only in photon flux density. Consequently, the R:FR ratio and other spectral quality parameters remained constant across treatments, preventing the activation of shade-avoidance morphogenetic programs that would otherwise induce dramatic architectural changes.

The small but significant increase in the number of immature leaves per plant in L300 and the higher dry mass of immature leaves in L300 and L400 suggest subtle adjustments in leaf production and turnover dynamics. These changes may reflect optimization of leaf appearance to match photosynthetic capacity with light availability. However, the overall stability of morphological traits across treatments indicates that *P. lanceolata* L. cv. “Ceres Tonic” exhibits relatively conservative morphological responses to moderate variations in light intensity under controlled environmental conditions.

### 3.2. Chlorophyll Fluorescence and Photosynthetic Adaptation

Although a statistically significant difference was detected in F_v_/F_m_ values in mature leaves, the changes of 1% lack biological importance [20,21]. Moreover, the absolute values remained well within the range of 0.75–0.85 considered indicative of optimal PSII activity and physiological comfort in non-stressed plants [22]. According to Kalaji et al. (2017) [22], F_v_/F_m_ values below 0.75 are generally associated with chronic photoinhibition or sustained photodamage to the reaction centers of PSII, whilst values above 0.80 reflect a fully functional photosynthetic apparatus. The fact that all treatments—in both mature and immature leaves—maintained F_v_/F_m_ above 0.83 indicates that none of the light intensities evaluated produce photoinhibition. The statistically significant reduction in F_v_/F_m_ observed in mature leaves at L400 (0.83 vs. 0.84) is therefore better interpreted as a subtle acclimatory downregulation of PSII efficiency—a well-documented response to moderately elevated irradiance—rather than evidence of photoinhibitory damage. Immature leaves, which showed higher and more stable F_v_/F_m_ values across all treatments (0.852–0.860), appear to be less sensitive to this acclimatory response, possibly reflecting greater metabolic plasticity in expanding tissues.

Notably, immature leaves reached reached An values comparable to those of mature leaves. This indicates a robust photosynthetic apparatus which is also supported for the maintenance of fluorescence parameters including F_0_, F_m_ and F_v_/F_m_. The strong correlation between A and gs in immature and mature leaves indicates the central role of stomatal conductance for photosynthesis, with the exception of 200 µmoles of light where irradiance becomes limiting for An. It should be noted, however, that these physiological observations characterize the stability of primary metabolism and do not, in themselves, provide mechanistic evidence regarding secondary metabolite biosynthesis.

The physiological stability observed across light treatments extends beyond leaf-level responses and is consistent with a broader pattern of morphological resilience documented in *P. lanceolata* L. under other environmental stressors. Furthermore, Merino et al. (2024a) [23] revealed changes in root architecture of *P. lanceolata* L. to acclimate and exhibit resilience to different defoliation intervals. Together with our light intensity data, these studies illustrate that *P. lanceolata* L. employs a hierarchical stress response: morphological/anatomical plasticity under severe or chronic stress (drought, temperature extremes, physical damage) and biochemical/physiological plasticity under moderate environmental variation (light intensity fluctuations). This hierarchical strategy minimizes carbon investment in structural changes whilst maintaining metabolic flexibility [24].

### 3.3. Light Intensity and Nutritional Quality

Intermediate light intensity (L300) produced herbage with superior nutritional quality compared to both lower (L200) and higher (L400) light intensities. Plants grown at L300 exhibited the lowest concentrations of NDF (44.4% DM) and ADF (17.4% DM), resulting in the highest metabolizable energy (11.8 MJ/kg DM). This non-linear response to light intensity aligns with established principles of forage quality regulation, where moderate environmental conditions balance photosynthetic carbon assimilation with minimized structural carbohydrate deposition. Our results are consistent with previous research demonstrating that sub-optimal light conditions can improve forage quality in temperate grass and legume species. Kephart and Buxton (1993) [25] reported that C3 perennial grasses grown at 37% of ambient sunlight exhibited 2–3% lower NDF concentrations and 3–5% higher *in vitro* digestible DM compared to full sunlight, concluding that stressful growth conditions limiting photosynthates may improve forage quality. Similarly, Lin et al. (2001) [26] found that 50% shade either maintained or slightly reduced NDF and ADF in shade-tolerant forages. More recently, Pang et al. (2019) [27] demonstrated that under moderate shade (45% of full sun), 14 of 22 forage species maintained a relative feed value equal to full sun while CP increased, confirming that intermediate light conditions optimize the trade-off between biomass production and nutritive value.

The mechanism underlying reduced fiber content at moderate light intensities involves the regulation of secondary cell wall biosynthesis by light-responsive transcriptional networks. Falcioni et al. (2018) [28] demonstrated that plants grown at 8.5% of sunlight exhibited lower lignin levels than those in full sunlight, establishing that high light fundamentally induces lignin biosynthesis and cell wall thickening. At the molecular level, Zhang et al. (2018) [29] showed that blue light regulates secondary cell wall thickening via MYC2/MYC4 activation of the NST1-directed transcriptional network in *Arabidopsis*, with lignin content being higher in cryptochrome 1 overexpressing plants. This light-induced increased deposition of structural cell wall components provides a mechanistic explanation for our observation that the highest light intensity (L400) resulted in elevated ADF concentrations, which include lignin as a major component.

The L300 treatment appears to represent an optimal threshold where photosynthetic carbon assimilation remains adequate for biomass production while avoiding the excessive activation of cell wall fortification pathways that characterize high-light acclimation. This interpretation is further supported by the work of Thorvaldsson and Andersson (2007) [30], who demonstrated that environmental stress factors, including excessive light, tend to decrease forage digestibility through increased fiber concentrations. In the context of *P. lanceolata*-based pasture systems, where nutritional quality directly influences animal performance and methane production, our findings suggest that moderate light availability, potentially achievable through strategic grazing management or silvopastoral systems, could enhance the nutritive value of *P. lanceolata* L. herbage while maintaining adequate biomass production.

### 3.4. Light Intensity and Secondary Metabolites

Although no statistically significant differences were detected in the concentrations of acteoside, aucubin, or catalpol in either immature or mature leaves (*p* > 0.05), a non-significant numerical trend toward higher acteoside concentrations in immature leaves at increasing light intensities was observed, which may warrant further investigation under more contrasting experimental conditions. The iridoid glycosides showed no consistent directional response across light treatments, suggesting that the intensity range evaluated (200–400 µmol photons m^−2^ s^−1^) was insufficient to substantially upregulate either the phenylpropanoid or the iridoid biosynthetic pathway. These findings contrast with previous studies reporting significant light-induced changes in *P. lanceolata* secondary metabolites under more extreme light differentials: Miehe-Steier et al. (2015) [14] reported that high light (175 µmol photons m^−2^ s^−1^) increased iridoid glycosides and verbascoside (acteoside) compared to low light (35 µmol photons m^−2^ s^−1^) in experimental grasslands. Specifically, leaf aucubin was approximately 4-fold higher under high light, catalpol was 2-fold higher, root iridoid glycosides were 7-fold higher, and verbascoside increased under high light but decreased under high nutrient availability. Similarly, Qu et al. (2021) [31] found that catalpol and aucubin concentrations were higher under high light in older *P. lanceolata* plants, with significant light × age interactions affecting SM accumulation. Although light intensity is recognized as a primary environmental driver of secondary metabolite biosynthesis in *P. lanceolata* L., with effects modulated by plant developmental stage and nutrient availability, the present results suggest that moderate variation within a relatively narrow range may not consistently alter secondary metabolite profiles.

### 3.5. Root Architectural Stability and Implications for Plant Persistence

The complete absence of light intensity effects on root traits, including total length, diameter, surface area, volume, branching architecture and biomass allocation, is remarkable given the well-documented responsiveness of *P. lanceolata* roots to other environmental variables. Merino et al. (2024b) [32] demonstrated that frequent intensive defoliation (every 10 days on average when fully extended leaves reached a length of 15 cm) drastically reduced total root length, root area and fine root density (<0.5 mm diameter), whilst simultaneously increasing allocation of high-molecular-weight non-soluble carbohydrate reserves to shoot regrowth. This stark contrast with our light intensity results reveals a fundamental principle: *P. lanceolata* root development is governed primarily by carbon availability and source–sink dynamics, not by light-mediated morphogenic signals per se.

Since An plateaued above 200 µmol photons m^−2^ s^−1^, as evidenced by invariant photosynthetic rates across treatments, all three light intensities provided sufficient photoassimilate to sustain normal root growth. Consequently, no compensatory adjustment in below-ground allocation was required. In contrast, defoliation creates an immediate carbon deficit by removing photosynthetic tissue, forcing mobilization of root non-soluble carbohydrate reserves and reallocation of current photosynthate to shoot regrowth at the expense of root maintenance [32]. The ecological interpretation is that *P. lanceolata* prioritizes root system integrity when carbon supply is adequate, regardless of light intensity, but sacrifices root growth only when carbon limitation is severe, as under intensive defoliation.

### 3.6. Implications for P. lanceolata-Based Pasture Management Under Climate Change

The integration of *P. lanceolata* L. into temperate pasture systems has gained considerable attention as a climate-smart strategy to enhance seasonal productivity, improve nutritional quality, and reduce the environmental footprint of grazing livestock [1,33,34]. Our findings contribute to this body of knowledge by demonstrating that light availability, a factor influenced by sward structure, grazing management, and silvopastoral design, can affect both the nutritional quality and physiological traits in *P. lanceolata* herbage.

The optimal fiber content and metabolizable energy achieved at 300 µmol photons m^−2^ s^−1^ suggest that *P. lanceolata* L. may benefit from moderate light conditions that balance photosynthetic productivity with minimized cell wall fortification. In mixed pastures, this could be achieved by managing sward density and companion grass competition to ensure adequate but not excessive light penetration to *P. lanceolata* rosettes. Overly dense swards that severely restrict light availability would limit photosynthesis and biomass production, while excessively open swards exposing *P. lanceolata* L. to full sunlight may trigger increased deposition of structural cell wall components that reduce digestibility. The capacity of *P. lanceolata* L. to maintain photosynthetic function across the range of light intensities evaluated (200–400 µmol photons m^−2^ s^−1^), as reflected by the absence of statistically significant differences in An and by F_v_/F_m_ values remaining above 0.83 across all treatments—indicative of functional, non-photoinhibited tissue —indicates a degree of physiological tolerance to moderate variation in light availability that should facilitate integration into diverse pasture systems.

With respect to secondary metabolites, a non-significant numerical trend toward higher acteoside concentrations in immature leaves at increasing light intensities was observed, but this pattern was not statistically supported and should not be used as a basis for management recommendations. Future studies employing a wider range of light intensities or larger sample sizes may help clarify whether light availability can consistently influence SM concentrations in *P. lanceolata* L. under field-relevant conditions. It is important to acknowledge that our study was conducted under controlled environmental conditions with stable temperature, humidity, and photoperiod, which do not fully replicate the dynamic field conditions experienced in pasture systems. Seasonal variations in air temperature, solar radiation, photoperiod, and plant phenological state are known to substantially influence SM concentrations in field-grown *P. lanceolata* L. [13,16]. Additionally, interactions with defoliation management, nutrient availability, and biotic stresses may modify the light intensity responses observed under controlled conditions. Nevertheless, our results provide valuable mechanistic insights into the direct effects of light intensity on *P. lanceolata* physiology and biochemistry, isolating these effects from confounding environmental variables and establishing a foundation for interpreting more complex field-based responses.

## 4. Materials and Methods

### 4.1. Experimental Site and Environmental Conditions

The experiment was conducted from July to October 2023 in controlled-environment growth chambers at the Animal Production Laboratory, Faculty of Agronomy, University of Concepción, Concepción, Chile. Three identical plant growth chambers (120 cm × 90 cm × 90 cm each) were used, with reflective interior surfaces to ensure uniform light distribution. Air temperature (20 ± 2 °C) and relative humidity (45–60%) were maintained through an air conditioning system and an extractor fan. These parameters were continuously monitored at 1 h intervals using thermochron data loggers (iButtons DS1923, Maxim Integrated Products Inc., San Jose, CA, USA) placed at plant height. Lighting was supplied by two high-efficiency 1000-watt LED panels (30 cm × 50 cm, ECO LED-1000, ProGarden, Dongguan, China) per chamber providing a constant spectral composition (68% red light, 12% blue light, 14% white light, 4% far-red light and 2% ultraviolet light), and a 14 h light: 10 h dark photoperiod. Photoperiod was maintained using a programmable digital timer (Sinotimer TM-615, Guangdong, China).

### 4.2. Experimental Design and Treatments

A completely randomized design was used, consisting of three light intensity treatments: 200, 300, and 400 µmol photons m^−2^ s^−1^ (L200, L300 and L400, respectively). This range is well-established in the controlled-environment literature; studies on forage grasses such as *L. perenne* and medicinal/aromatic herbs such as *Ocimum basilicum* have employed the same intensity levels to evaluate physiological, growth, and biochemical responses under LED illumination [35,36]. Each treatment was replicated eight times, resulting in 24 pots as experimental units. Each pot contained four *P. lanceolata* plants, resulting in 32 plants per treatment, and 96 plants overall. To ensure uniformity, pots were matched based on the height and weight of mature non-senescent leaves per plant, averaging 20.1 cm of leaves height and 2.32 cm of leaves width per plant. Subsequently, the pots were randomly assigned to the treatments.

### 4.3. Plant Establishment and Maintenance

*P. lanceolata* seeds (cultivar “Ceres Tonic”) were germinated in 10 cm Petri dishes under controlled conditions. After one week, seedlings with radicles measuring 5–20 mm were transplanted into a 50-cell plant nursery tray (10 cm in height) to promote early root development. Four weeks later, four uniform seedlings were carefully selected and transplanted into plastic pots (20 cm × 20 cm × 25 cm, 7 L capacity). Pots were filled with 1 kg of a substrate composed of 80% peat (Kekkila Professional, Vantaa, Finland), 10% perlite, and 10% vermiculite. After one month, all pots were thoroughly irrigated, and their saturated weights were recorded following the methodology described by Earl (2003) [37] to establish an irrigation protocol. This process was repeated two months later to accommodate the increase in pot weight due to root biomass accumulation. The pots were weighed weekly using a precision balance (DY208, BEL Engineering, Monza, Italy, 30 kg capacity and 0.5 g accuracy), and water was added as needed to maintain field capacity at approximately 330 mL per pot. To ensure adequate nutrient availability, plants were fertilized weekly with Plant Food Phostrogen (14% N, 16% P_2_O_5_, 18% K_2_O, plus trace elements) at 2.8 g L^−1^. These management practices were strictly followed throughout the entire experiment. Light intensity levels were adjusted by modifying the distance between the LED panels and the pot surface to achieve the target photosynthetically active radiation (PAR) for each treatment. PAR was monitored weekly using a quantum sensor (SQ-521, Apogee Hansatech Intruments, Logan, UT, USA) to ensure uniform light distribution throughout the experiment. To minimize the effects of spatial variability in light distribution within the chambers, pots were rotated 90° and repositioned every four days within the chambers. Plants were defoliated every 15 days to a residual height of 5 cm once they had developed at least six fully expanded leaves (approximately three months after planting), following established protocols for *P. lanceolata* L. under controlled conditions to simulate frequent grazing and enable regrowth assessment [32].

### 4.4. Variables Measured

#### 4.4.1. Morphological Characteristics and Dry Biomass Composition Across the Cuts

Every week, the length and width of a mature fully extended non-senescent mature leaf (mature leaf) per plant were measured (32 leaves per treatment, 96 leaves in total). Leaf height was measured from the base to the top of the leaf, while the leaf width was defined as the maximum lateral distance between the two leaf margins. Both measurements were taken using a millimeter ruler in four plants per pot.

Every three weeks, the herbage of each pot was manually harvested at 5 cm of residual height using scissors for characterization of morphological traits of above-ground biomass. Before the harvest, the following variables were recorded for each plant: total number of leaves, number of mature leaves, number of immature leaves, number of senescent (dead) leaves, and the number of reproductive stems. In addition, the total number of leaves and reproductive stems was counted per pot. Subsequently, the forage harvested was separated into leaves, reproductive stems, and dead material and dried in a forced-air oven (BOV-V125F, Biobase, Jinan, China) at 60 °C until a constant weight was reached. The dry weight (g DM) of each component was recorded to determine its proportional contribution of each fraction to total DM biomass above 5 cm.

#### 4.4.2. Chlorophyll Fluorescence Measurements in Mature and Immature Leaves

Chlorophyll fluorescence parameters, such as the minimum chlorophyll fluorescence (F_0_), maximum chlorophyll fluorescence (F_m_), and the maximum quantum yield (F_v_/F_m_) of photosystem II (PSII), were assessed weekly using a JUNIOR-PAM fluorometer (Heinz Walz GmbH, Effeltrich, Germany) between each cut. These parameters were recorded in two leaf types per plant: mature and immature leaves. Before measurements, each selected leaf was dark-adapted for at least 45 min. Fluorescence was then measured by placing the glass fiber on the middle third of the dark-acclimated leaf surface. One reading was taken per leaf, and four measurements per leaf type were obtained per pot. The average of these readings was used to generate a single representative value per immature and mature leaves (32 leaves per treatment per leaf type, 96 leaves in total per leaf type).

#### 4.4.3. Chemical Composition and Water-Soluble Carbohydrate Analysis

Composite herbage samples (approximately 100 g of dried biomass each) were collected from each pot, representing all herbage collected from the three harvests conducted throughout the experimental period. These samples also incorporated forage from the final destructive harvest, thereby capturing the complete temporal profile of above-ground biomass accumulation. The samples were ground, sieved through a 1 mm mesh and stored for chemical analysis. A total of 24 composite samples (eight per treatment) were analyzed. The DM, N and ash concentrations were determined following the methods of the Association of Official Analytical Chemists (AOAC; 2001.12, 2001.11, and 942.05, respectively [38]). Crude protein (CP) concentration was estimated using the Kjeldahl method, applying a conversion factor of 6.25. Neutral detergent fiber (NDF) and acid detergent fiber (ADF) concentrations were analyzed according to Van Soest (1991) [39] and AOAC (2006) [40], respectively. Metabolizable energy (ME) was estimated using the equation provided by the National Research Council [41]. Composite samples of 25 mg of oven-dried forage collected during the experimental period from each pot, including the material collected at the final harvest, were used to determine the water-soluble carbohydrate (WSC) concentrations. The material was previously homogenized and ground to 1 mm using a forage grinder. The phenol–sulfuric acid colorimetric method [42] was used for WSC quantification. WSCs were extracted with 80% ethanol-distilled water solution in a 15 mL plastic tubes. The resulting WSC extracts were then separated into low-molecular-weight fractions (e.g., glucose, fructose, sucrose, and short-chain fructans) and high-molecular-weight fractions (e.g., long-chain fructans). Raffinose pentahydrate (≥99% purity; Sigma-Aldrich, St. Louis, MO, USA) was used as the calibration standard, as raffinose represents the primary carbohydrate reserve in *P. lanceolate* [43]. Absorbance was measured at 490 nm using a 96-well microplate in a Synergy H1M microplate reader (BioTek, Santa Clara, USA). Results were expressed as milligrams of raffinose equivalents per gram of DM (mg g^−1^ DM).

#### 4.4.4. Destructive Harvested Measurements

At the end of the experimental period, a destructive harvest was carried out to evaluate the effects of light intensity on DM allocation, morphological and physiological traits, and chemical composition of *P. lanceolata* plants. Before the destructive harvest was undertaken, morphophysiological assessments were conducted on all experimental units. To quantify leaf area, one mature and one immature leaf were collected from the same individual plant in each pot to ensure measurement consistency. Leaves were excised at the base of the lamina and immediately scanned at 1400 dpi using a flatbed scanner (Epson L3250, model C634H, Nagano, Japan). The resulting digital images were analyzed using Image J software version 1.54e (NIH, Bethesda, MD, USA) to determine leaf area, following standardized image processing protocols [44]. For morphological composition, the following parameters were recorded for each pot: the number of plant individuals and seedlings, total number of live leaves, number of mature leaves, number of immature leaves, and the number of reproductive stems and shoots. Additionally, the number of shoots, immature leaves, residual leaves and reproductive stems was recorded for each plant.

Gas exchange measurements were also conducted using an infrared gas analyzer (LI-6400XT, LI-COR Inc., Lincoln, NE, USA) following the methodology described by Bascuñán-Godoy et al. (2018) [45]. Net photosynthetic rate (An), stomatal conductance (g_s_), and apparent transpiration rate (E) were assessed on two leaf types per plant: a mature fully expanded non-senescent leaf and an immature leaf. Measurements were taken on the third leaf from the top after a 10 min acclimation period under a photosynthetic photon flux density (PPFD) of 1500 µmol photons m^−2^ s^−1^.

A mature fully expanded non-senescent leaf and an immature leaf were collected from each plant, freeze-dried, and ground into a fine powder for SM analysis. For each sample, 0.1 g of tissue was extracted with 10 mL of methanol (MeOH, 99%, Sigma-Aldrich) in 15 mL centrifuge tubes. Samples were incubated in a shaking water bath at 70 °C for 30 min, with intermittent agitation every 5 min. After centrifugation at 15,000 rpm for 10 min, the supernatant was filtered through a 0.45 µm PVDF filter and stored at −20 °C until further analysis. Subsequently, an aliquot of 2 mL of the supernatant was diluted with 8 mL of Type I water and passed through a 0.2 µm syringe filter (Whatman International Ltd., Maidstone, England) for high-performance liquid chromatography (HPLC) analysis. Catalpol and aucubin were simultaneously quantified using 10 µL of the diluted extract, while acteoside was analyzed directly from the undiluted extract after 0.2 µm syringe filtration. Quantification of SMs was performed using a Hitachi HPLC-DAD system (Hitachi Technologies, Merck, Germany) equipped with a Kromasil C18 reversed-phase column (250 mm × 4.6 mm, particle size 5 µm; Nouryon, Göteborg, Sweden). The mobile phases consisted of 2% acetonitrile in water for catalpol and aucubin, and 29% MeOH in water containing 1% formic acid for acteoside. The flow rate was 1 mL min^−1^. Detection wavelengths were set to 240 nm for catalpol and aucubin, and 330 nm for acteoside (Tamura and Nishibe, 2002) [13]. Calibration was performed using commercial standards: catalpol and aucubin (Sigma-Aldrich, USA) and acteoside (ChromaDex, Longmont, CO, USA). Each sample was analyzed in triplicate, and results were expressed in milligrams of each metabolite per 100 g of DM (mg 100 g^−1^ DM).

All above-ground plant material was harvested (destructive harvest) using hand shears and separated into leaves, reproductive stems, and dead tissue (senescent leaves and stems) to determine their relative contribution to total DM. Below-ground DM, including roots and crown, was carefully collected, washed with 20% chlorine bleach solution, and manually retrieved using a 1 mm sieve. Both above- (shoots, live leaves, live stems, roots, and dead material comprising dead leaves and reproductive stems) and below-ground samples were dried in a forced-air oven (BOV V-125F, BioBase, Jinan, China) at 60 °C until constant weight and weighed to determine total DM and the DM allocation. The dry weights of the above-ground mass components were used to determine their relative contribution to total herbage DM.

For root trait analysis, dried root samples from four plants per pot were scanned using a high-resolution scanner (Epson Expression 12000XL, Nagano, Japan) equipped with dual light sources to minimize root overlap. Roots were submerged in a 1 cm water layer in a transparent tray (30 cm × 20 cm) and scanned at 200 dpi (dots per inch). Scanned images were subsequently analyzed using WinRHIZO Reg software (v7.0, Regent Instruments Inc., Quebec, QC, Canada) to obtain total root length, average root diameter, root surface area, root volume, and architectural parameters as the number of tips, forks (root bifurcations), and crossings (overlapping root sections) [32].

### 4.5. Statistical Analysis

Datasets were analyzed using analysis of variance (ANOVA) after verifying normality and homogeneity of variance assumptions. The SMs did not meet the normality and homoscedasticity assumptions; therefore, they were analyzed with a Kruskal–Wallis one-way ANOVA. Linear Pearson’s correlation coefficient (r) was estimated to examine the relationship between A and gs of mature and immature leaves. Differences between treatment means were evaluated using Tukey’s test at a 95% confidence level (*p* ≤ 0.05).

## 5. Conclusions

This study shows that variation in light intensity within the tested range (200–400 µmol photons m^−2^ s^−1^) had limited effects on morphology, biomass production, root architecture, and secondary metabolite concentrations in *Plantago lanceolata* L., indicating a high degree of physiological stability. Specifically, moderate light (300 µmol photons m^−2^ s^−1^) optimizes nutritional quality by minimizing fiber content and maximizing metabolizable energy, whereas higher light intensity (400 µmol photons m^−2^ s^−1^) induces subtle photoprotective adjustments in chlorophyll fluorescence parameters of mature leaves, with no statistically significant changes detected in secondary metabolite concentrations. The capacity of *P. lanceolata* L. to maintain stable photosynthetic functions and stores of bioactive compounds across the evaluated range of light intensities reflects notable phenotypic and metabolic stability. These findings suggest that moderate variation in light availability can influence forage quality without substantially altering plant growth or metabolism. From a practical perspective, light conditions modulated through pasture management may contribute to improving forage nutritive value, as light availability can be influenced by sward structure and grazing management. However, further research is needed to assess the consistency of these responses across a wider range of environmental conditions.

## Figures and Tables

**Table 1:** Chlorophyll fluorescence and gas exchange parameters of *Plantago lanceolata* L. under different light intensities (200, 300, and 400 µmol photons m^−2^ s^−1^).

	Light Intensity (µmol Photons m^−2^ s^−1^)					
Variable	200	300	400	SEM	F-Value	*p*-Value
F_0_ mature leaf	122 a	116 b	119 ab	0.001	4.356	0.027
F_m_ mature leaf	781 a	717 b	737 b	7.50	9.787	0.001
F_v_/F_m_ mature leaf	0.84 a	0.84 a	0.83 b	0.001	4.151	0.030
F_0_ immature leaf	100	86.3	95.9	7.21	0.978	0.399
F_m_ immature leaf	686	634	656	43.6	0.356	0.707
F_v_/F_m_ immature leaf	0.86	0.85	0.86	0.0045	0.596	0.563
An mature leaf (µmol CO_2_ m^−2^ s^−1^)	15.4	11.5	12.7	3.11	0.40	0.678
gs mature leaf (mmol H_2_O m^−2^ s^−1^)	230	189	258	46.9	0.55	0.584
E mature leaf (mmol H_2_O m^−2^ s^−1^)	3.25	3.11	3.51	0.429	0.22	0.801
An immature leaf (µmol CO_2_ m^−2^ s^−1^)	9.54	9.61	11.38	1.021	1.04	0.372
gs immature leaf (mmol H_2_O m^−2^ s^−1^)	231	139	271	46.5	2.11	0.146
E immature leaf (mmol H_2_O m^−2^ s^−1^)	3.08	2.44	3.79	0.459	2.17	0.139

ab: Different letters within a row under the light intensity columns indicate differences in mean values (*p* ≤ 0.05). SEM: standard error of the mean. F_0_: minimum chlorophyll fluorescence. F_m_: maximum chlorophyll fluorescence. F_v_/F_m_: maximum quantum yield of PSII, An: net photosynthetic rate, gs: stomatal conductance, and E: apparent transpiration rate.

**Table 2:** Pearson correlations between net photosynthetic rate (An) and stomatal conductance (gs) in *Plantago lanceolata* L. grown at different light intensity (200, 300, and 400 µmol photons m^−2^ s^−1^) in mature (ML) and immature (IL) leaves.

PAR (µmol Photon m^−2^ s^−1^)	Leaf Type	r (An–gs)	*p*-Value
200	ML	−0.34	0.420
200	IL	0.72	0.045
300	ML	0.90	0.0026
300	IL	0.92	0.0011
400	ML	0.87	0.0047
400	IL	0.81	0.014

Pearson correlation coefficient (r) and associated *p*-value. *n* = 8 leaves per light intensity × leaf type.

**Table 3:** Chemical composition and water-soluble (WSC) concentration in *Plantago lanceolata* L. grown under three light intensity levels (200, 300 and 400 µmol photons m^−2^ s^−1^).

	Light Intensity					
Variable	200	300	400	SEM	F Value	*p*-Value
Dry matter concentration (g 100 g^−1^ FM)	21.6	23.6	22.6	1.45	3.3784	0.0615
Crude protein (% DM)	9.48	7.82	10.56	0.664	3.2413	0.0671
Neutral detergent fiber (% DM)	48.8 ab	44.4 b	59.7 a	2.66	6.5659	0.0090
Acid detergent fiber (% DM)	20.9 a	17.4 b	22.8 a	0.64	13.7395	0.0004
ME (MJ kg^−1^ DM)	11.4 b	11.8 a	11.2 b	0.08	13.7395	0.0004
Ash (% DM)	10.67	9.33	10.00	0.579	2.7403	0.0888
Total carbohydrates (mg of raffinose 100 g^−1^ DM)	133	147	160	0.4	0.5550	0.5855
Low-WSC (mg of raffinose 100 g^−1^ DM)	94.9	122.2	123.8	13.33	1.1166	0.3531
High-WSC (mg of raffinose 100 g^−1^ DM)	38.2	25.3	36.1	4.60	1.7058	0.2150

ab: Different letters within a row under the light intensity columns indicate differences in mean values (*p* ≤ 0.05). SEM: standard error of the mean. FM: fresh matter. ME: metabolizable energy.

**Table 4:** Plant morphological characteristics of *Plantago lanceolata* L. at final destructive harvest under three light intensity levels (200, 300, and 400 µmol photons m^−2^ s^−1^).

	Light Intensity					Overall Average
Variable	200	300	400	SEM	*p*-Value	
Number of plants pot^−1^	13.8	13.8	11.6	1.08	0.294	13.0
Number of seedlings pot^−1^	2.88	2.12	1.25	0.986	0.517	2.08
Number of shoots pot^−1^	9.75	9.75	7.62	1.077	0.294	9.04
Number of immature leaves pot^−1^	21.6	28.1	22.1	2.26	0.100	23.9
Number of residual leaves pot^−1^	1.88	1.88	1.38	0.486	0.706	1.71
Number of shoots pot^−1^	1.25	1.5	1.38	0.389	0.902	1.38
Number of mature leaves in the main plants pot^−1^	14.0	13.4	14.4	0.73	0.627	13.9
Total number of leaves pot^−1^	64.4	72.9	56.5	5.30	0.117	64.6
Number of reproductive stems in the main plants pot^−1^	7.88	7.38	10.25	1.389	0.315	8.50
Number of reproductive stems in shoots pot^−1^	1.88	1.50	2.12	0.816	0.863	1.83
Total number of reproductive stems pot^−1^	9.75	8.88	12.38	1.677	0.327	10.3
Number of shoots plant^−1^	2.44	2.44	1.91	0.269	0.294	2.26
Number of immature leaves plant^−1^	2.16 c	2.94 b	2.34 a	0.155	0.005	2.48
Number of residual leaves plant^−1^	0.781	0.844	0.688	0.1593	0.786	0.771
Number of reproductive stems plant^−1^	2.44	2.22	3.09	0.419	0.327	2.58

abc: Different letters within a row under the light intensity columns indicate differences in mean values (*p* ≤ 0.05). SEM: standard error of the mean. Plants: Refers to the original transplanted individuals. Seedlings: Refers to spontaneously germinated secondary plants. Shoots: Refers to plants produced by the original plants.

**Table 5:** Dry matter allocation to above- and below-ground plant components of *Plantago lanceolata* L. at final destructive harvest under three light intensity levels (200, 300, and 400 µmol photons m^−2^ s^−1^).

	Light Intensity					Overall Average
Variable	200	300	400	SEM	*p*-Value	
Dry mass of immature leaves > 5 cm (g DM)	0.49	0.537	0.651	0.0674	0.245	0.559
Proportion of immature leaves > 5 cm (% DM > 5 cm)	18.6	20.8	19.2	0.64	0.075	19.5
Dry mass of immature leaves < 5 cm (g DM)	0.257 b	0.419 a	0.361 a	0.0265	0.001	
Proportion of immature leaves < 5 cm (% DM < 5 cm)	16.9	18.4	21.5	2.27	0.359	18.9
Total dry mass of immature leaves (g DM)	0.747	0.956	1.012	0.0815	0.075	0.905
Dry mass of mature leaves > 5 cm (g MS)	3.15	3.76	2.75	0.335	0.125	3.22
Proportion of dry mass of mature leaves > 5 cm (% DM > 5 cm)	20.1	22.4	22.1	0.69	0.061	21.6
Dry mass of mature leaves < 5 cm (g DM)	1.291	1.554	1.24	0.1265	0.195	1.36
Total dry mass of mature leaves (g DM)	4.44	5.32	3.99	0.447	0.128	4.58
Total dry mass of green leaves (immature and mature leaves) (g DM)	5.19	6.27	5	0.452	0.126	5.49
% Total DM of green leaves (immature and mature leaves)	295	331	251	23.0	0.069	292
Reproductive stems DM > 5 cm (g MS)	1.31	1.15	1.76	0.315	0.386	1.41
Reproductive stems DM < 5 cm (g MS)	0.17	0.189	0.25	0.044	0.416	0.203
Reproductive stems DM (g MS/pot^−1^)	1.49	1.34	2.01	0.356	0.393	1.61
% of reproductive stems DM > 5 cm (%)	24.1	25.8	23.7	1.29	0.484	24.5
% of reproductive stems DM < 5 cm (%)	22.3	24.2	22.0	0.65	0.052	22.9
% Total of reproductive stems DM	594	659	1342	279.1	0.135	865
Dry mass senescent leaves > 5 cm	0.496	0.714	0.659	0.3699	0.456	0.623
Dry mass senescent leaves < 5 cm	1.81	2.29	1.86	0.535	0.145	1.99
Dry mass senescent leaves (g DM pot^−1^)	2.31 b	3.01 a	2.52 ab	0.562	0.048	
% Dry mass senescent leaves > 5 cm	29.3	27.5	29.2	1.50	0.654	28.7
% Dry mass senescent leaves < 5 cm	54.9	50.3	48.8	3.11	0.369	51.3
% Total dry mass senescent leaves	25.9	28.8	26.7	1.99	0.578	27.1
Total aerial biomass (g DM)	8.99	10.62	9.53	0.598	0.169	9.71
Primary root dry mass pot^−1^ (g DM/pot^−1^)	0.268	0.199	0.201	0.0235	0.082	0.223
Secondary root dry mass (g DM/pot^−1^)	1.53	1.71	1.86	0.148	0.294	1.70
Total dry mass roots pot^−1^ (g DM/pot^−1^)	1.80	1.91	2.07	0.151	0.462	1.92
Aerial biomass/root biomass	0.260	5.670	4.930	0.6630	0.663	3.62

ab: Different letters within a row under the light intensity columns indicate differences in mean values (*p* ≤ 0.05). SEM: standard error of the mean. Overall means are shown only when no significant differences were detected. Immature leaves: leaves that have not yet reached full expansion. Mature leaves: fully expanded leaves that have reached their final dimensions.

## Data Availability

The data presented in this study are available on request from the corresponding author, because they are part of an ongoing study.
